# Hypokinetic Activity of Menthofuran on the Gastrointestinal Tract in Rodents

**DOI:** 10.1155/2023/2726794

**Published:** 2023-06-10

**Authors:** Railson de Sousa Santos, Paulo Humberto Moreira Nunes, Geovanni de Morais Lima, Ana Karolinne da Silva Brito, James Frederico Rocha Pacheco, Heldemys da Costa Medina, Maria Ivone Mendes Benigno, Damião Pergentino de Sousa, Oseas Florêncio de Moura-Filho, Francisco Valmor Macedo Cunha, Renandro de Carvalho Reis, Rita de Cássia Meneses Oliveira, Daniel Dias Rufino Arcanjo, Maria do Carmo de Carvalho e Martins

**Affiliations:** ^1^Department of Biophysics and Physiology, Federal University of Piauí, PI, Teresina, Brazil; ^2^Department of Morphology, Federal University of Piauí, PI, Teresina, Brazil; ^3^Department of Pharmaceutical Sciences, Federal University of Paraíba, PB, João Pessoa, Brazil; ^4^Physios-Functional Health Clinic Ltd., Piauí, Teresina, Brazil; ^5^Uninovafapi University Centre, PI, Teresina, Brazil

## Abstract

The acute toxicity and hypokinetic activity induced by menthofuran on the gastrointestinal tract of rodents were investigated in the present study. An absence of acute toxicity was observed. Menthofuran delayed gastric emptying at oral doses of 25, 50, and 100 mg/kg in the experimental model of phenol red, as well as it reduced the intestinal transit at oral doses of 50 and 100 mg/kg. Interestingly, a scopolamine-similar hypokinetic effect was observed for menthofuran. In the experimental model of castor oil-induced intestinal hypermotility, menthofuran (50 and 100 mg/kg) reduced the number of loose stools as observed for the normal group. Additionally, menthofuran induced a marked concentration-dependent relaxation in rat ileum segments precontracted with KCl (EC_50_ = 0.059 ± 0.008 *μ*g/mL) or carbachol (EC_50_ = 0.068 ± 0.007 *μ*g/mL). These results suggest the possible decrease of calcium influx underlying the effects of menthofuran on the gastrointestinal tract, which opens the door for further study regarding this potential application for the treatment of gastrointestinal disorders, noting possible limitations of its use due to adverse effects in children.

## 1. Introduction

Diarrhea is a clinical condition characterized by the increased passage of loose stools associated with an increased number of stools, usually more than three bowel movements per day, and is an important public health issue [1]. In 2015, this condition was the cause of death of 499,000 children, and 688 million children experiencing pain [2], most of them in developing countries most of them with a low human development index (HDI) [3].

Diarrhea treatment usually involves the administration of oral rehydration therapy (ORT) and in some cases, antibiotics directed against a particular pathogen [4]. However, pharmacological therapy against gastrointestinal disorders of motility and secretion may be indicated, as antispasmodic drugs with antisecretory activity and can alleviate the manifestations of the diarrheal process, reducing dehydration and pain and preventing death [5]. Despite their widespread use, these drugs may have multiple side effects, among them, effects on the central nervous system, such as addiction [6], and on the intestinal tract, such as paralytic ileus [7].

Traditional medical practices using medicinal plants or their isolated compounds have represented a promising alternative for the treatment of gastrointestinal disorders, especially in view of the numerous side effects associated with conventional therapy. In this sense, terpenes, a class of plant secondary metabolites have attracted pharmacological interest because some substances of this class have demonstrated gastroprotective [8], anti-inflammatory [9, 10], antioxidant [11], and antimicrobial [12] effects.

The essential oil of peppermint is obtained by steam distillation from the fresh leaves of *Mentha x piperita* L., an herbaceous plant of the *Lamiaceae* family native from Europe that has great importance in agriculture, medicine, pharmaceutical industry and biotechnology [13, 14]. According to the Committee for Herbal Medicinal Products of the European Medicines Agency (EMA/HMPC) in the EMA Herbal Monograph [15]; this essential oil (peppermint oil) has well-established use as an herbal medicinal product for the symptomatic relief of minor spasms of the gastrointestinal tract, flatulence, and abdominal pain, especially in patients with irritable bowel syndrome. Besides, it is a traditional herbal medicinal product used for the relief of symptoms in coughs and colds. However, its use is contraindicated in children under 2 years of age, because menthol can induce reflex apnoea and laryngospasm.

The monoterpenes menthol (35–45%) and menthone (15–20%) are the major constituents in the essential oil of *Mentha x piperita* L., and menthofuran (2–7%) is one of the other present components [16, 17]. Menthol had its antispasmodic [18, 19] and gastroprotective [20] activities previously investigated, whereas isomenthone showed protective activity against tumor necrosis factor-*α*–induced cell death [21]. Interestingly, a previous study has reported the menthofuran-induced gastroprotective effect by underlying antioxidant mechanisms [22]. Besides, since any study regarding the effects of menthofuran in the gastrointestinal motility, the present study aimed to investigate menthofuran-induced effects in the gastrointestinal motility by using *in vitro* and *in vivo* approaches.

## 2. Materials and Methods

### 2.1. Animals

Male Wistar rats (180–200 g) and Swiss mice (20–30 g) were obtained from the Animal Facility of the Federal University of Piauí; they were maintained under temperature-controlled conditions (24 ± 2°C), 12 h/12 h light/dark cycle, and received food and water ad libitum. All procedures followed the ethical principles of the National Council of Animal Experimentation Control (CONCEA). The project was approved by the Ethics Committee on the Use of Animals from of the Federal University of Piauí (CEUA/UFPI) (protocol No. 086/2015).

### 2.2. Chemical and Drugs

Menthofuran, scopolamine, castor oil, carboxymethyl cellulose, activated charcoal, and carbachol were purchased from Sigma–Aldrich (St. Louis, MO, United States of America); loperamide, from Janssen-Cilag, LTDA (Brazil); phenol red and trichloroacetic acid, from Reagen Quimibrás Chemical Industries SA (Brazil). Menthofuran was solubilized in 1.0% Tween 80: distilled water (v/v); scopolamine in distilled water, and phenol red in 2% carboxymethylcellulose solution, each prepared for immediate use.

### 2.3. Evaluation of the Acute Oral Toxicity in Mice

Acute toxicity was evaluated according to the protocol 420 (fixed dose method) of the Organization for Economic Cooperation and Development (OECD, 2001). Briefly, male Swiss mice (20–30 g) were randomly divided into two groups (*n* = 6/group). The control and treatment groups were orally treated with a single dose of vehicle (1.0% Tween 80) and menthofuran (2,000 mg/kg), respectively. Animals were observed for up to 8 hours on the first day of treatment, and daily thereafter for 14 days. Afterwards, the following clinical and behavioral parameters were evaluated: alertness, sedation, ptosis, dyspnea, urination, diarrhea, convulsions, spontaneous motor activity, postural reflex, piloerection, response to touch, and number of deaths.

### 2.4. Gastric Emptying

The gastric emptying and small intestinal transit were assessed by the phenol red content assay, modified from the method described by Izbeki et al. [23]. Briefly, Wistar male rats (180–200 g) were housed in metabolic cages for four days and kept in a solid fast for 18 hours preceding the experiment. On the day of the experiment, groups of rats (7 animals/group) were treated orally with water vehicle (1% Tween 80, 5 mL/kg), menthofuran (25, 50, and 100 mg/kg), or scopolamine (10 mg/kg). One hour after, animals were orally treated with 1.5 mL of a liquid food consisting of 0.5 mg/mL phenol red diluted in 2.0% carboxymethylcellulose solution. After 20 minutes, they were euthanized. After laparotomy, cardia, pylorus, and ileocecal junction were ligated, and then small intestine and stomach were removed and separated. Each part was fragmented and placed in 50 mL of 0.1 N NaOH and kept in a water bath at 80°C under stirring for one hour. Samples of 5 mL of the suspensions were mixed with 20% trichloroacetic acid solution (0.5 mL) following by centrifugation at 3,000 rpm for 10 min. Next, supernatants (1.0 mL) were mixed with 0.5 N NaOH (2.0 mL). The optical density of phenol red was read at 560 nm (Biospectro SP-220, EQUIP Ltda., Curitiba, Brazil) using distilled water as blank. Gastric retention (GE) was calculated by the ratio between the amount of dye obtained from the small intestine and the total amount found in both segments. Results were expressed as a percentage.

### 2.5. Intestinal Transit

Male Swiss mice (20–30 g) were housed in metabolic cages for four days, and then they were subjected to fasting food, but not water, in the 18 hours preceding the experiment. The animals were randomly distributed into 5 group of eight animals each and treated orally with the vehicle (1% Tween 80, 0.1 mL/10 g) or menthofuran (25, 50, and 100 mg/kg) or scopolamine (12 mg/kg). Thirty minutes after treatments, the animals received an aqueous suspension of 2.5% activated charcoal in 3% carboxymethylcellulose solution (0.1 mL/10 g) and, after another 30 minutes they were euthanized. Then, the pylorus was ligated and the stomach and the small intestine of each animal were removed and distended. The intestinal transit was evaluated by determining the distance traveled by the charcoal in 30 min from the pylorus until the last portion of the intestine containing at least 1 cm of continuous charcoal and expressed as a percentage of the total length of the small intestine.

### 2.6. Intestinal Hypermotility-Induced by Castor Oil

Groups of male Swiss mice (20–40 g) were individually maintained in standard conditions in metabolic cages to prevent coprophagy for at least 4 days. The chow, but not water, was removed 18 hours before the experiments, and the animals randomized into six groups of eight animals. The animals of the sham and diarrhea control groups were treated orally with water and castor oil (0.1 mL/animal), respectively. After 30 minutes, they orally received vehicle (1.0% Tween 80, 0.2 mL/10 g). The next four groups were orally treated with menthofuran (25, 50, and 100 mg/kg) and loperamide (6 mg/kg). Then, the animals were individually observed for 4 hours, and the excretion of watery stools after 30, 60, and 90 min and 2, 3, and 4 hours was count in order to evaluate the onset and severity of the diarrhea induced by castor oil.

### 2.7. Spasmolytic Activity in Ileum

Wistar albino rats (180–200 g) were housed in metabolic cages for four days and then they were subjected to fasting food, but not water, in the 18 hours preceding the experiment. On the day of experiment, animals were euthanized, and the distal ileus were removed and placed on Petri plates with modified Krebs solution and aerated with carbogenic mixture (95% O_2_ and 5% CO_2_). Ileum segments (2-3 cm) were maintained in modified Krebs solution and coupled to a force transducer connected to an acquisition system (AECAD 1604 AQCAD 2.0.5., AVS Project, SP, Brazil), following to stabilization for 30 minutes. Subsequently, two contractions of similar magnitudes were induced by addition of 1 *μ*M carbachol or 40 mM KCl (or decreasing KCl concentration to 40 mM) in different preparations. After the second stable tonic contraction induced by the contractile agents, menthofuran was added in increasing and cumulative concentrations for checking its spasmolytic effect in the contracted ileum. The values of EC_50_ (concentration at which a substance induces half of the maximum effect) of menthofuran required to induce relaxation of the ileum in the presence of carbachol or KCl were expressed as mean ± standard error of the mean in *μ*g/mL of the resulting values of each experiment.

### 2.8. Statistical Analysis

The results were presented as mean ± S.E.M. Significance analyses gastric emptying, intestinal transit and intestinal hypermotility protocols were performed by one-way variance analysis (ANOVA) followed by the Tukey's or Newman–Keuls' test. The EC_50_ values for spasmolytic activity on ileum isolated protocols were calculated by nonlinear regression and the means were compared by the unpaired the Student “*t*” test. Data were analyzed using Prism version 5.0 software (GraphPad Software, La Jolla, CA, USA). Differences were considered significant when *p* < 0.05.

## 3. Results

### 3.1. Acute Toxicity

After administration of a single oral dose of menthofuran at 2000 mg/kg, no deaths or clinical and behavioral alterations were observed for 14 days. These results indicate the absence of systemic acute toxicity and considering the absence of deaths, the median lethal dose (LD_50_) for menthofuran was not determined.

### 3.2. Effect of Menthofuran on the Gastric Emptying in Rats

**Figure 1:**
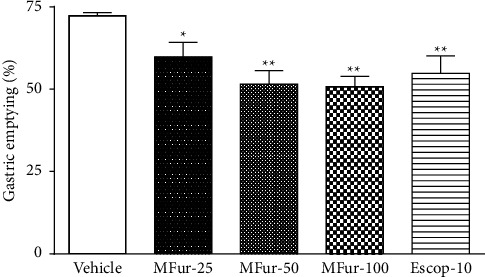
Effect of oral administration of menthofuran 25 mg/kg (MFur-25), 50 mg/kg (MFur-50) and 100 mg/kg (MFur-100) or scopolamine 10 mg/kg (Escop-10) on the gastric emptying in rats (*n* = 8/group). Data are presented as the mean ± S.E.M. ^*∗*^*p* < 0.05 and ^*∗∗*^*p* < 0.01 when compared with the control group (vehicle). ANOVA followed by the Tukey's test.

In the gastric emptying model using phenol red method, the vehicle group showed an emptying mean (%) of 72.3 ± 0.9. The menthofuran at doses of 25, 50 and 100 mg/kg (MFur-25: 59.8 ± 4.4; MFur-50: 51.6 ± 4.1; and MFur-100: 50.8 ± 3.1), orally administered, was able to significantly reduce the gastric emptying (*p* < 0.001) compared to the vehicle group. The effects in the gastric emptying of the three tested doses of menthofuran were similar to the effect of scopolamine 10 mg/kg (54.8 ± 5.3%) used as a standard pharmacological drug (Figure 1).

### 3.3. Effect of Menthofuran on Small Intestinal Transit in Mice

**Figure 2:**
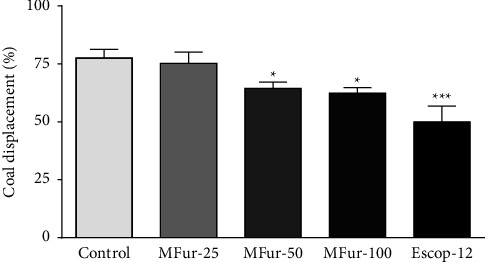
Effect of menthofuran 25 mg/kg (MFur-25), 50 mg/kg (MFur-50) and 100 mg/kg (MFur-100) or scopolamine 12 mg/kg (Escop-12) on the small intestinal transit of an aqueous suspension of charcoal in mice (*n* = 8/group). Data are presented as the mean ± S.E.M. ^*∗*^*p* < 0.05 and ^*∗∗∗*^*p* < 0.001 when compared with the control group (vehicle) (ANOVA followed by the Tukey's test).

In the evaluation of the effect of menthofuran on small intestinal transit in mice (Figure 2), it was observed that the menthofuran-treated groups (50 and 100 mg/kg, p.o.) had significantly reduced gut transit (*p* < 0.05) compared with the control group. The group treated with scopolamine also had significantly reduced intestinal transit (50.0 ± 6.8; *p* < 0.001) compared with the control group (77.6 ± 3.6), but not when compared with the groups treated with menthofuran (MFur-50: 64.5 ± 2.6 and MFur-100: 62.4 ± 2.4). The effect of menthofuran was similar for the two tested doses.

### 3.4. Effect of Menthofuran on Castor Oil-Induced Diarrhea

Diarrhea was observed half an hour after castor oil administration and lasted four hours. Menthofuran (100 mg/kg) and loperamide (12 mg/kg) significantly reduced total elimination of watery stools during the 4-hour observation time (Table 1).

**Figure 3:**
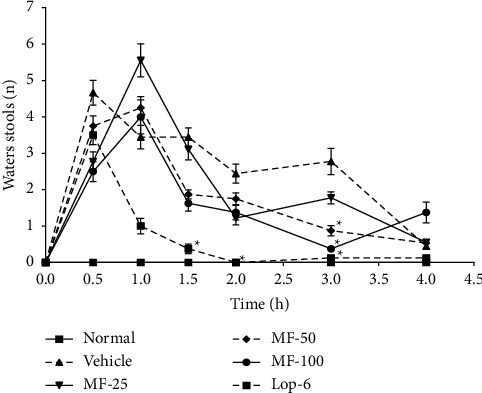
Effect of the oral treatment with menthofuran 25 mg/kg (MFur-25), 50 mg/kg (MFur-50), and 100 mg/kg (MFur-100) or loperamide 6 mg/kg (Lop-6) on the elimination of fluid stools during the 4-hour observation time diarrheal activity of castor oil in mice.

In all the treated groups' diarrhea was settled half an hour after castor oil administration. In the loperamide-treated group, diarrhea was completely inhibited after one hour and thirty minutes. Diarrhea total inhibition also occurred in menthofuran-treated groups at the doses of 50 and 100 mg/kg, but only after the third hour of observation (Figure 3).

To evaluate the effect of menthofuran (25, 50 and 100 mg/kg, p.o.) in the model of diarrhea induced by castor oil, significant reduction was observed in the number of watery stools eliminated in groups treated with menthofuran at 50 and 100 mg/kg (p.o.) in the third time (Figure 3) when compared with the vehicle group (diarrheal), but not compared to the control group (nondiarrheal). The default group (loperamide 6 mg/kg, p.o.) on the other hand, fecal excretion was significantly lower when compared to the vehicle group from the first hour after treatment but did not differ statistically (*p* > 0.05) compared to experimental treatment (menthofuran 100 mg/kg, p.o.), at the third hour.

### 3.5. Spamolitic Effect of Menthofuran on Isolated Ileum

**Figure 4:**
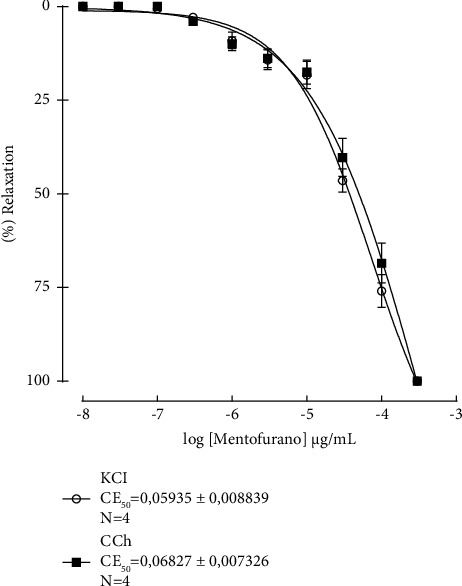
Effect of menthofuran (10^−8^ to 10^−3^ *μ*g/mL) on tonic contraction induced by KCl (40 mM) and carbachol (10^−6^ M in isolated ileum of rats (*n* = 4)). The symbols represent the percentage of the average ± standard error of the mean.

Menthofuran at a range concentration of 10^−8^–10^−3^ *μ*g/mL (*n* = 4) induced a significant concentration-dependent relaxation on ileum preparations precontracted by KCl or carbachol (Figure 4). In relation to the potency, menthofuran showed similar potencies for relaxation of the ileum, no significant differences (*p* < 0.05) were observed between the concentrations required to promote relaxation of KCl (EC_50_ = 0.059 ± 0.008 *μ*g/mL or carbachol-contracted ileum (EC_50_ = 0.068 ± 0.007 *μ*g/mL).

## 4. Discussion

This study investigated the effect of menthofuran on the gastrointestinal tract, aiming to provide additional evidence of the medicinal use of *Mentha x piperita* L. or its components in the treatment of gastrointestinal disorders. In evaluating the motility of the gastrointestinal tract, gastric emptying and gut motility models were used, as well as the castor oil-induced diarrhea model which demonstrated that menthofuran at doses of 50 and 100 mg/kg (p.o.) reduced the gastric emptying and the intestinal transit and delayed the on-set and reduced the duration of the diarrhea induced by castor oil, thus an presenting antidiarrheal effect. In an *in vitro* study, menthofuran, in a dose-dependent manner, was able to promote relaxation of the ileum precontracted by KCl or carbachol.

As the gastrointestinal peristalsis is regulated by the release of the neurotransmitter acetylcholine acting on muscarinic receptors and promoting peristalsis, it was suggested that menthofuran could act in three different ways: (1) as an antagonist of muscarinic receptors subtype M3, resulting in reduction of the acetylcholine activity on the gastrointestinal tract and thereby reducing peristalsis; (2) as an agonist of opioid receptors, acting on *μ* gastrointestinal receptors, resulting in inhibition of adenosine monophosphate (AMP) transformation to cyclic adenosine monophosphate (cyclic AMP) and subsequent reduction of the release of the neurotransmitter acetylcholine and inhibiting intestinal peristalsis [24]; or (3) as a voltage-sensitive Ca^2+^ channels blocker, resulting in inhibition of smooth muscle contraction, since the opening of these channels are the most important event to complete depolarization and subsequent contraction of intestinal muscles [25, 26]. Hiki et al. [27] found that the peppermint essential oil (PEO) was able to reduce gastric spasms during upper gastrointestinal endoscopy. Asao et al. [28] evaluated the effectiveness of PEO in reducing colon spasms during colonoscopy and found a spasmolytic effect in 88.5% of treated patients, representing an alternative to the conventional use of cholinergic blockers during this procedure. In a pharmacodynamic study, Goerg and Spilker [29] showed that PEO promoted reduction of gut transit and relaxation of the gallbladder in healthy subjects. On the other hand, Alam et al. [30], evaluating the efficacy of PEO in patients with irritable bowel syndrome suffering from diarrhea, observed a reduction of pain but no other symptoms. And Merat et al. [31] conducted a randomized, double-blind, placebo-controlled clinical trial in which they showed the effect of peppermint oil in relieving abdominal pain or discomfort and the severity of symptoms when compared to placebo.

The use of the castor oil-induced diarrhea model relies in the fact that, when hydrolyzed in the gut, the oil generates an active metabolite, ricinoleic acid, which promotes inflammation of the intestinal mucosa and secretion of water, as well as altered permeability of the small intestine to electrolytes, resulting in increased intestinal motility and, thereby, diarrhea [32]. The results of the present study suggest that menthofuran has antidiarrheal activity in rodents, since it was observed that at doses of 50 and 100 mg/kg (p.o.), menthofuran was able to control the establishment of diarrhea, in a dose-dependent manner, reducing the number of loose stools when compared to the control group (vehicle), and thus the severity of diarrhea.

The antidiarrheal effect of menthofuran can be explained by reduced water and electrolyte secretion or by alteration of gastrointestinal peristalsis. Rozza et al. [33], evaluating the effect of menthol in castor oil-induced diarrhea model, observed that menthol showed a similar effect compared with loperamide in reducing the number of loose stools, thus presenting antidiarrheal effect. Bustos-Brito et al. [34] found that a thymol derivative compound, a monoterpene, isolated from *Agerantina cylindrica*, showed inhibitory activity against hyperperistaltic movements induced by charcoal associated with gum Arabic in rats. Negi et al. [35] found that the methanolic extract of the essential oil of *Saussurea lappa* C., which has sesquiterpenes as its major constituents, has antidiarrheal activity comparable to diphenoxylate in a castor oil-induced diarrhea model.

After performing the *in vivo* study, menthofuran activity on intestinal peristalsis was evaluated using a tonic contraction *in vitro* model with ileum of rats, in order to identify the possible mechanism of its spasmolytic effect. In our study, menthofuran, in a dose-dependent manner, was able to promote relaxation of the ileum precontracted by KCl or carbachol similarly. KCl, a depolarizing agent, when added to the tub, produces contraction of segments of the ileum through changes in the electrochemical potential to K^+^, once the K^+^ concentration is higher in the extracellular space, which results in depolarization of the cell membrane by increasing the opening of K^+^ channels and Ca^2+^ channels (voltage-dependent), thus acting as an electromechanical agent promoting contraction [36]; whereas carbachol, which is an agonist of muscarinic receptors subtype M3, widely distributed in the ileum, binds to its receptor, which is coupled to a G protein, and promotes the production of second messengers DAG and IP3, resulting in the release of Ca^2+^ from sarcoplasmic reticulum and Golgi complex, besides recruiting extracellular Ca^2+^ [37], promoting ileum contraction. By observing that menthofuran had a similar potency to the tonic contraction induced by KCl and carbachol, we hypothesized the involvement of a common pathway, resulting in blocking Ca^2+^ entry into the cell through Ca^2+^ channels (voltage-dependent) and therefore inhibiting contractile response to contractile agents tested. However, further studies are required for the complete elucidation of the mechanisms underlying the spasmolytic effect of menthofuran.

Hills and Aaronson [38] investigated the mechanism of action of peppermint essential oil (PEO) in guinea pig large intestine and observed relaxation of smooth musculature by reducing calcium influx. Hawthorn et al. [39], evaluating the effect of menthol and PEO in isolated ileum and heart preparations, found that smooth musculature relaxation was due to antagonism of Ca^2+^ channels. Blanco et al. [40]; evaluating the antispasmodic effects of essential oils of two different chemotypes of *Lippia alba* M., found evidence that the mechanism of action of the spasmolytic effect was probably due to reduced Ca^2+^ influx or activation of the contractile processes mediated by Ca^2+^. Accordingly, in a study that investigated the effects induced by menthol on the human distal colon mechanical activity *in vitro* and to analyze the mechanism of action, Amato et al. [41] demonstrated that menthol, the major constituent of peppermint oil, induces spasmolytic effects in human colon circular muscle inhibiting directly the gastrointestinal smooth muscle contractility, through the block of Ca^2+^ influx through sarcolemma L-type Ca^2+^ channels.

Peppermint oil is composed primarily of menthol and menthone and other possible constituents include pulegone, menthofuran, and limone (however, the content of secondary metabolites in Peppermint leaves (*Mentha x piperita* L.) kept under long-day conditions contain menthol, menthone, and only traces of menthofuran, while the plants grown under short-day conditions contain menthofuran as a major component of the volatile oil [42]. (+)-pulegone is a central intermediate in the biosynthesis of (−)-menthol and, depending on environmental conditions, this branch point metabolite may be reduced by pulegone reductase (PR) to (−)-menthone route to menthol, or oxidized by menthofuran synthase (MFS) to (+)-menthofuran [43]. (6R)-(+)-Menthofuran, a minor constituent of peppermint oil, is a major metabolite of (1R)-(+)-*β*-pulegone [44] and the metabolic fate of (+)-pulegone is controlled through transcriptional regulation of MFS and menthofuran influences, directly or indirectly, this process by downregulating transcription from PR and/or decreasing PR message stability [43].

Regarding the toxicity of menthofuran, studies report a high toxicity associated with metabolites produced by the biotransformation of pulegone and menthofuran, product of its metabolism, which are capable of covalently binding to cellular proteins and reduce the levels of enzymes in the cytochrome P450 superfamily, which metabolize xenobiotics, resulting in hepatotoxicity and death [45, 46], and for this reason the acute toxicity test was conducted. Following administration of an oral single dose of menthofuran (2,000 mg/kg, p.o.) no signs of toxicity were observed, which brought increased safety to its use in the present study. Thorup et al. [47] evaluating the effects of administration, over 28 days, at doses of 0 to 160 mg/kg/day (p.o.) of pulegone, a precursor of menthofuran, observed that higher doses were capable of inducing stasis, body weight changes and histopathological changes in the liver and white matter of the rat cerebellum. Baibars et al. [48] reported that chronic exposure to menthol, one of the monoterpenes isolated from mint (*Mentha piperita* L.), resulted in the skin (macular skin lesions), neurological (dizziness and loss of consciousness), and gastrointestinal (diarrhea) disorders. However, Khalil et al. [49] showed that peppermint essential oil had a protective effect against carbon tetrachloride-induced liver injury, increasing the activity of antioxidant enzymes superoxide dismutase and glutathione, as well as reducing the levels of malondialdehyde, a marker of cell damage produced by oxidative stress.

As for the safety of using pulegone and menthofuran, rats were the most sensitive species in toxicity studies and the available data suggest a comparable pattern of response to high pulegone exposure of rats and humans regarding the potential generation of reactive metabolites, acute kidney and liver toxicity and glutathione depletion as the basis for determining a threshold for toxicity. Thus, the value of 37.5 mg/kg bw per day, based in the 3-month rat repeat-dose toxicity study, was taken as a NOAEL (No Observed Adverse Effect Level) value and by the using an uncertainty factor of 50, as a recommendation, the acceptable exposure limit proposed by the EMA/HMPC would be 0.75 mg/kg bw per day in humans [50].

## 5. Conclusion and Outlook

The analysis of the results indicates that menthofuran did not induce an acute toxic effect in the single-dose acute toxicity study and decreases gastrointestinal motility, which was demonstrated by delayed gastric emptying, reduction of the intestinal transit, and spasmolytic effect in ileum segments. The spasmolytic activity against KCl and carbachol-induced contractions may indicate involvement of inhibition of voltage-dependent Ca^2+^ channels. These findings indicate the therapeutic potential of menthofuran to be used as an antidiarrheal and d spasmolytic drug. However, the current literature available is limited, and further studies *in vitro* and *in vivo* are needed to fully understand its mechanism of action and to generate consistent evidence for conducting clinical trials to assess the quality, safety, and therapeutic efficacy of this natural product.

## Figures and Tables

**Table 1:** Effect of the oral treatment of mice with menthofuran 25 mg/kg (MFur-25), 50 mg/kg (MFur-50), and 100 mg/kg (MFur-100) or loperamide 6 mg/kg (Lop-6) on the elimination of watery stools on diarrheal activity of castor oil model.

Treatment	*n*	Dose	Diarrheal faeces	Diarrheal
			(*n*/4 h; mean ± S.E.M.)	Inhibition (%)
Normal	8	—	0.13 ± 0.13	—
Control	8	10 mL/kg	17.31 ± 1.94	0.0
Menthofuran	8	25 mg/kg	15.00 ± 1.04	13.3
	8	50 mg/kg	12.93 ± 1.15	24,1
	8	100 mg/kg	11.36 ± 2.07^*∗*^	34.4
Loperamide	8	6 mg/kg	4.79 ± 0.75^*∗∗∗*^	72.3

Data are presented as the mean ± S.E.M. ^*∗∗∗*^*p* < 0.001, ^*∗*^*p* < 0.05 when compared with the control group (ANOVA and the Newman–Keuls' test).

## Data Availability

The data used to support the findings of this study are available from the corresponding author upon reasonable request.
